# FINDINGS FROM THE ALZHEIMER’S POETRY PROJECT LONG-TERM CARE IMPLEMENTATION STUDY

**DOI:** 10.1093/geroni/igz038.715

**Published:** 2019-11-08

**Authors:** Daniel B Kaplan, Gary Glazner

**Affiliations:** 1 Adelphi University, Garden City, New York, United States; 2 The Alzheimer's Poetry Project, New York, New York, United States

## Abstract

Dementia interventions grounded in group participation in the cultural arts (e.g., poetry, storytelling, music, and dance) have been growing in reach in recent decades. Yet this growth has been stymied by a lack of empirical evidence to demonstrate measurable outcomes. In the current IRB-approved research study, the Alzheimer’s Poetry Project (APP), in partnership with 15 Wisconsin nursing homes, provided staff training in an innovative non-pharmacological intervention for people living with dementia. The goal of APP is to facilitate creative self-expression, social and intellectual stimulation, respectful acceptance, validation of personhood, and valued inclusion of people living with Alzheimer's disease and other dementias through the performance and creation of poetry. APP’s proven track record at over 500 facilities in 34 states and six countries internationally, serving over 40,000 people worldwide, demonstrates APP’s ability to bring high-quality creative arts programming to people living with dementia. Through the implementation of APP in three groups of five nursing homes in Wisconsin, activities and care staff were trained to deliver the intervention over three-month periods. After training was completed, researchers evaluated and compared APP to non-arts programming in the same facilities. A novel dementia arts mapping technique was created to document observable impacts of interventions on social engagement, alertness, vocalization, vocabulary, affect, and self-expression. 35 interventions were documented, and variability in participant experiences reveal significantly greater impacts on personhood and creativity during the 11 APP interventions than during the 24 non-arts interventions. Implications for promoting dementia care programming grounded in group participatory arts will be discussed.

